# Who Shares? Who Doesn't? Factors Associated with Openly Archiving Raw Research Data

**DOI:** 10.1371/journal.pone.0018657

**Published:** 2011-07-13

**Authors:** Heather A. Piwowar

**Affiliations:** Department of Biomedical Informatics, University of Pittsburgh, Pittsburgh, Pennsylvania, United States of America; Science and Technology Facilities Council, United Kingdom

## Abstract

Many initiatives encourage investigators to share their raw datasets in hopes of increasing research efficiency and quality. Despite these investments of time and money, we do not have a firm grasp of who openly shares raw research data, who doesn't, and which initiatives are correlated with high rates of data sharing. In this analysis I use bibliometric methods to identify patterns in the frequency with which investigators openly archive their raw gene expression microarray datasets after study publication.

Automated methods identified 11,603 articles published between 2000 and 2009 that describe the creation of gene expression microarray data. Associated datasets in best-practice repositories were found for 25% of these articles, increasing from less than 5% in 2001 to 30%–35% in 2007–2009. Accounting for sensitivity of the automated methods, approximately 45% of recent gene expression studies made their data publicly available.

First-order factor analysis on 124 diverse bibliometric attributes of the data creation articles revealed 15 factors describing authorship, funding, institution, publication, and domain environments. In multivariate regression, authors were most likely to share data if they had prior experience sharing or reusing data, if their study was published in an open access journal or a journal with a relatively strong data sharing policy, or if the study was funded by a large number of NIH grants. Authors of studies on cancer and human subjects were least likely to make their datasets available.

These results suggest research data sharing levels are still low and increasing only slowly, and data is least available in areas where it could make the biggest impact. Let's learn from those with high rates of sharing to embrace the full potential of our research output.

## Introduction

Sharing and reusing primary research datasets has the potential to increase research efficiency and quality. Raw data can be used to explore related or new hypotheses, particularly when combined with other available datasets. Real data are indispensable for developing and validating study methods, analysis techniques, and software implementations. The larger scientific community also benefits: Sharing data encourages multiple perspectives, helps to identify errors, discourages fraud, is useful for training new researchers, and increases efficient use of funding and population resources by avoiding duplicate data collection.

Eager to realize these benefits, funders, publishers, societies, and individual research groups have developed tools, resources, and policies to encourage investigators to make their data publicly available. For example, some journals require the submission of detailed biomedical datasets to publicly available databases as a condition of publication [1], [2]. Many funders require data sharing plans as a condition of funding: Since 2003, the National Institutes of Health (NIH) in the USA has required a data sharing plan for all large funding grants [3] and has more recently introduced stronger requirements for genome-wide association studies [4]. As of January 2011, the US National Science Foundation requires that data sharing plans accompany all research grant proposals [5]. Several government whitepapers [6], [7] and high-profile editorials [8], [9] call for responsible data sharing and reuse. Large-scale collaborative science is increasing the need to share datasets [10], , and many guidelines, tools, standards, and databases are being developed and maintained to facilitate data sharing and reuse [12], [13].

Despite these investments of time and money, we do not yet understand the impact of these initiatives. There is a well-known adage: You cannot manage what you do not measure. For those with a goal of promoting responsible data sharing, it would be helpful to evaluate the effectiveness of requirements, recommendations, and tools. When data sharing is voluntary, insights could be gained by learning which datasets are shared, on what topics, by whom, and in what locations. When policies make data sharing mandatory, monitoring is useful to understand compliance and unexpected consequences.

Dimensions of data sharing action and intention have been investigated by a variety of studies. Manual annotations and systematic data requests have been used to estimate the frequency of data sharing within biomedicine [14], [15], [16], [17], though few attempts were made to determine patterns of sharing and withholding within these samples. Blumenthal [18], Campbell [19], Hedstrom [20], and others have used survey results to correlate self-reported instances of data sharing and withholding with self-reported attributes like industry involvement, perceived competitiveness, career productivity, and anticipated data sharing costs. Others have used surveys and interviews to analyze opinions about the effectiveness of mandates [21] and the value of various incentives [20], [22], [23], [24]. A few inventories list the data-sharing policies of funders [25], [26] and journals [1], [27], and some work has been done to correlate policy strength with outcome [2], [28]. Surveys and case studies have been used to develop models of information behavior in related domains, including knowledge sharing within an organization [29], [30], physician knowledge sharing in hospitals [31], participation in open source projects [32], academic contributions to institutional archives [33], [34], the choice to publish in open access journals [35], sharing social science datasets [20], and participation in large-scale biomedical research collaborations [36].

Although these studies provide valuable insights and their methods facilitate investigation into an author's intentions and opinions, they have several limitations. First, associations to an investigator's intention to share data do not directly translate to associations with actually sharing data [37]. Second, associations that rely on self-reported data sharing and withholding likely suffer from underreporting and confounding, since people admit withholding data much less frequently than they report having experienced the data withholding of others [18].

I suggest a supplemental approach for investigating research data-sharing behavior. I have collected and analyzed a large set of observed data sharing actions and associated study, investigator, journal, funding, and institutional variables. The reported analysis explores common factors behind these attributes and looks at the association between these factors and data sharing prevalence.

I chose to study data sharing for one particular type of data: biological gene expression microarray intensity values. Microarray studies provide a useful environment for exploring data sharing policies and behaviors. Despite being a rich resource valuable for reuse [38], microarray data are often, but not yet, universally shared. Best-practice guidelines for sharing microarray data are fairly mature [12], [39]. Two centralized databases have emerged as best-practice repositories: the Gene Expression Omnibus (GEO) [13] and ArrayExpress [40]. Finally, high-profile letters have called for strong journal data-sharing policies [41], resulting in unusually strong data sharing requirements in some journals [42]. As such, the results here represent data sharing in an environment where it has been particularly encouraged and supported.

## Methods

In brief, I used a full-text query to identify a set of studies in which the investigators generated gene expression microarray datasets. Best-practice data repositories were searched for associated datasets. Attributes of the studies were used to derive factors related to the investigators, journals, funding, institutions, and topic of the studies. Associations between these study factors and the frequency of public data archiving were determined through multivariate regression.

### Studies for analysis

The set of “gene expression microarray creation” articles was identified by querying the title, abstract, and full-text of PubMed, PubMed Central, Highwire Press, Scirus, and Google Scholar with portal-specific variants of the following query:

**(“gene expression” [text] AND “microarray” [text] AND “cell” [text] AND “rna” [text])**

**AND (“rneasy” [text] OR “trizol” [text] OR “real-time pcr” [text])**

NOT (“tissue microarray*” [text] OR “cpg island*” [text])

Retrieved articles were mapped to PubMed identifiers whenever possible; the union of the PubMed identifiers returned by the full text portals was used as the definitive list of articles for analysis. An independent evaluation of this approach found that it identified articles that created microarray data with a precision of 90% (95% confidence interval, 86% to 93%) and a recall of 56% (52% to 61%), compared to manual identification of articles that created microarray data [43].

Because Google Scholar only displays the first 1000 results of a query, I was not able to view all of its hits. I tried to identify as many Google Scholar search results as possible by iteratively appending a variety of attributes to the end of the query, including various publisher names, journal title words, and years of publication, thereby retrieving distinct subsets of the results 1000 hits at a time.

### Data availability

The dependent variable in this study was whether each gene expression microarray research article had an associated dataset in a best-practice public centralized data repository. A community letter encouraging mandatory archiving in 2004 [41] identified three best-practice repositories for storing gene expression microarray data: NCBI's Gene Expression Omnibus (GEO), EBI's ArrayExpress, and Japan's CIBEX database. The first two were included in this analysis, since CIBEX was defunct until recently.

An earlier evaluation found that querying GEO and ArrayExpress with article PubMed identifiers located a representative 77% of all associated publicly available datasets [44]. I used the same method for finding datasets associated with published articles in this study: I queried GEO for links to the PubMed identifiers in the analysis sample using the “pubmed_gds [filter]” and queried ArrayExpress by searching for each PubMed identifier in a downloaded copy of the ArrayExpress database. Articles linked from a dataset in either of these two centralized repositories were considered to have “shared their data” for the endpoint of this study, and those without such a link were considered not to have shared their data.

### Study attributes

For each study article I collected 124 attributes for use as independent variables. The attributes were collected automatically from a wide variety of sources. Basic bibliometric metadata was extracted from the MEDLINE record, including journal, year of publication, number of authors, Medical Subject Heading (MeSH) terms, number of citations from PubMed Central, inclusion in PubMed subsets for cancer, whether the journal is published with an open-access model and if it had data-submission links from Genbank, PDB, and SwissProt.

ISI Journal Impact Factors and associated metrics were extracted from the 2008 ISI Journal Citation Reports. I quantified the content of journal data-sharing policies based on the “Instruction for Authors” for the most commonly occurring journals.

NIH grant details were extracted by cross-referencing grant numbers in the MEDLINE record with the NIH award information (http://report.nih.gov/award/state/state.cfm). From this information I tabulated the amount of total funding received for each of the fiscal years from 2003 to 2008. I also estimated the date of renewal by identifying the most recent year in which a grant number was prefixed by a “1” or “2” —indication that the grant is “new” or “renewed,” respectively.

The corresponding address was parsed for institution and country, following the methods of Yu et al. [45]. Institutions were cross-referenced to the SCImago Institutions Rankings 2009 World Report (http://www.scimagoir.com/) to estimate the relative degree of research output and impact of the institutions.

Attributes of study authors were collected for first and last authors (in biomedicine, customarily, the first and last authors make the largest contributions to a study and have the most power in publication decisions). The gender of the first and last authors were estimated using the Baby Name Guesser website (http://www.gpeters.com/names/baby-names.php). A list of prior publications in MEDLINE was extracted from Author-ity clusters, 2009 edition [46], for the first and last author of each article in this study. To limit the impact of extremely large “lumped” clusters that erroneously contain the publications of more than one actual author, I excluded prior publication lists for first or last authors in the largest 2% of clusters and instead considered these data missing. For each paper in an author's publication history with PubMed identifiers numerically less than the PubMed identifier of the paper in question, I queried to find if any of these prior publications had been published in an open source journal, were included in the “gene expression microarray creation” subset themselves, or had reused gene expression data. I recorded the date of the earliest publication by the author and the number of citations to date that their earlier papers received in PubMed Central.

I attempted to estimate if the paper itself reused publicly available gene expression microarray data by looking for its inclusion in the list that GEO keeps of reuse at http://www.ncbi.nlm.nih.gov/projects/geo/info/ucitations.html.

Data collection scripts were coded in Python version 2.5.2 (many libraries were used, including EUtils, BeautifulSoup, pyparsing and nltk [47]) and SQLite version 3.4. Data collection source code is available at github (http://github.com/hpiwowar/pypub).

### Statistical methods

Statistical analysis was performed in R version 2.10.1 [48]. P-values were two-tailed. The collected data were visually explored using Mondrian version 1.1 [49] and the Hmisc package [50]. I applied a square-root transformation to variables representing count data to improve their normality prior to calculating correlations.

To calculate variable correlations, I used the hector function in the polycor library. This computes polyserial correlations between pairs of numeric and ordinal variables and polychoric correlations between two ordinal variables. I modified it to calculate Pearson correlations between numeric variables using the rcorr function in the Hmisc library. I used a pairwise-complete approach to missing data and used the nearcor function in the sfsmisc library to make the correlation matrix positive definite. A correlation heatmap was produced using the gplots library.

I used the nFactors library to calculate and display the scree plot for correlations.

Since the correlation matrix was not well-behaved enough for maximum-likelihood factor analysis, first-order exploratory factor analysis was performed with the fa function in the psych library, using the minimum residual (minres) solution and a promax oblique rotation. Second-order factor analysis also used the minres solution but a varimax rotation, since I wanted these factors to be orthogonal. I computed the loadings on the original variables for the second-order factors using the method described by Gorsuch [51].

Before computing the factor scores for the original dataset, missing values were imputed through Gibbs sampling with two iterations through the mice library.

Using this complete dataset, I computed scores for each of the datapoints onto all of the first and second-order factors using Bartlett's algorithm as extracted from the factanal function. I submitted these factor scores to a logistic regression using the lrm function in the rms package. Continuous variables were modeled as cubic splines with 4 knots using the rcs function from the rms package, and all two-way interactions were explored.

Finally, hierarchical supervised clustering on the datapoints was performed to learn which factors were most predictive and then estimated the data sharing prevalence in a contingency table of these two clusters split at their medians.

## Results

Full-text queries for articles describing the creation of gene expression microarray datasets returned PubMed identifiers for 11,603 studies.

MEDLINE fields were still “in process” for 512 records, resulting in missing data for MeSH-derived variables. Impact factors were found for all but 1,001 articles. Journal policy variables were missing for 4,107 articles. The institution ranking attributes were missing for 6,185. I cross-referenced NIH grant details for 3,064 studies (some grant numbers could not be parsed, because they were incomplete or strangely formatted). I was able to determine the gender of the first and last authors, based on the forenames in the MEDLINE record, for all but 2,841 first authors and 2,790 last authors. All but 1,765 first authors and 797 last authors were found to have a publication history in the 2009 Author-ity clusters.

PubMed identifiers were found in the “primary citation” field of dataset records in GEO or ArrayExpress for 2,901 of the 11,603 articles in this dataset, indicating that 25% (95% confidence intervals: 24% to 26%) of the studies deposited their data in GEO or ArrayExpress and completed the citation fields with the primary article PubMed identifier. This is my estimate for the prevalence of gene expression microarray data deposited into the two predominant, centralized, publicly accessible databases.

**Figure 1:**
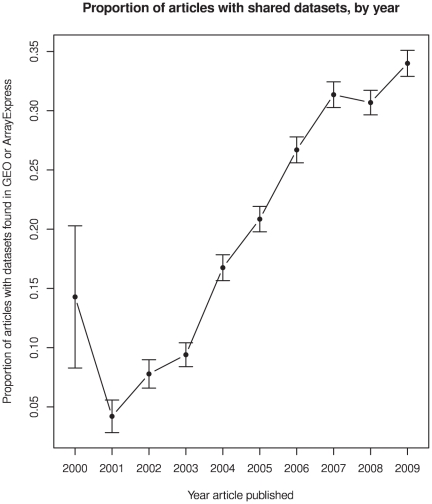
Proportion of articles with shared datasets, by year (error bars are 95% confidence intervals of the proportions).

This data-sharing rate increased with each subsequent article publication year, as seen in Figure 1, increasing from less than 5% in 2001 to 30%–35% in 2007–2009. Accounting for the sensitivity of my automated method for detecting open data anywhere on the internet (about 77% [44]), it could be estimated that approximately 45% (0.35/0.77) of recent gene expression studies have made their data publicly available.

**Figure 2:**
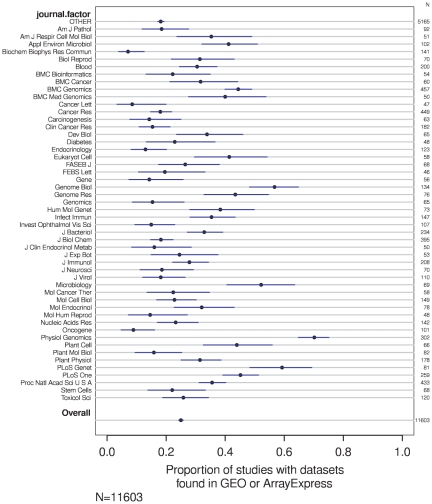
Proportion of articles with shared datasets, by journal (error bars are 95% confidence intervals of the proportions).

The data-sharing rate also varied across journals. Figure 2 shows the data-sharing rate across the 50 journals with the most articles in this study. Many of the other attributes were also associated with the prevalence of data sharing in univariate analysis, as seen in Figures S1, S2, S3, S4, S5.

### First-order factors

I tried to use a scree plot to determine the optimal number of factors for first-order analysis. Since the scree plot did not have a clear drop-off, I experimented with a range of factor counts near the optimal coordinates index (as calculated by nScree in the nFactors R-project library) and finalized on 15 factors. The correlation matrix was not sufficiently well-behaved for maximum-likelihood factor analysis, so I used a minimum residual (minres) solution. I chose to rotate the factors with the promax oblique algorithm, because first-order factors were expected to have significant correlations with one another. The rotated first-order factors are given in Table 1 with loadings larger than 0.4 or less than −0.4. Some of the loadings are greater than one. This is not unexpected since the factors are oblique and thus the loadings in the pattern matrix represent regression coefficients rather than correlations. Correlations between attributes and the first-order factors are given in the structure matrix in Table S1. The factors have been named based on the variables they load most heavily, using abbreviations for publishing in an Open Access journal (OA) and previously depositing data in the Gene Expression Omnibus (GEO) or ArrayExpress (AE) databases.

**Table 1:** First-order factor loadings.

Large NIH grant
0.97 num.post2005.morethan1000k.tr
0.96 num.post2005.morethan750k.tr
0.92 num.post2004.morethan750k.tr
0.91 num.post2004.morethan1000k.tr
0.91 num.post2005.morethan500k.tr
0.89 num.post2006.morethan1000k.tr
0.89 num.post2006.morethan750k.tr
0.86 num.post2004.morethan500k.tr
0.85 num.post2006.morethan500k.tr
0.84 num.post2003.morethan750k.tr
0.84 num.post2003.morethan1000k.tr
0.80 num.post2003.morethan500k.tr
0.74 has.U.funding
0.71 has.P.funding
0.58 nih.sum.avg.dollars.tr
0.56 nih.sum.sum.dollars.tr
0.44 nih.max.max.dollars.tr
Has journal policy
1.00 journal.policy.contains..geo.omnibus
0.95 journal.policy.at.least.requests.sharing.array
0.95 journal.policy.mentions.any.sharing
0.93 journal.policy.contains.word.microarray
0.91 journal.policy.requests.sharing.other.data
0.85 journal.policy.says.must.deposit
0.83 journal.policy.contains.word.arrayexpress
0.72 journal.policy.requires.microarray.accession
0.71 journal.policy.requests.accession
0.58 journal.policy.contains.word.miame.mged
0.48 journal.microarray.creating.count.tr
0.45 journal.policy.mentions.consequences
0.42 journal.policy.general.statement
NOT institution NCI or intramural
0.59 pubmed.is.funded.non.us.govt
0.55 institution.is.higher.ed
−0.89 institution.nci
−0.86 pubmed.is.funded.nih.intramural
−0.42 country.usa
Count of R01 & other NIH grants
1.15 has.R01.funding
1.14 has.R.funding
0.89 num.grants.via.nih.tr
0.86 nih.cumulative.years.tr
0.82 num.grant.numbers.tr
0.80 max.grant.duration.tr
0.66 pubmed.is.funded.nih
0.50 nih.max.max.dollars.tr
0.45 num.nih.is.nigms.tr
0.44 country.usa
0.42 has.T.funding
0.41 num.nih.is.niaid.tr
Journal impact
0.88 journal.5yr.impact.factor.log
0.88 journal.impact.factor.log
0.85 journal.immediacy.index.log
0.70 journal.policy.mentions.exceptions
0.54 journal.num.articles.2008.tr
0.51 journal.policy.contains.word.miame.mged
−0.61 journal.policy.contains.word.arrayexpress
−0.48 pubmed.is.open.access
Last author num prev pubs & first year pub
0.84 last.author.num.prev.pubs.tr
0.74 last.author.year.first.pub.ago.tr
0.73 last.author.num.prev.pmc.cites.tr
0.68 last.author.num.prev.other.sharing.tr
0.48 country.japan
0.44 last.author.num.prev.microarray.creations.tr
Journal policy consequences & long half-life
0.78 journal.policy.mentions.consequences
0.73 journal.cited.halflife
0.60 pubmed.is.bacteria
0.42 journal.policy.requires.microarray.accession
−0.54 pubmed.is.open.access
−0.45 journal.policy.general.statement
Institution high citations & collaboration
0.76 institution.mean.norm.citation.score
0.72 institution.international.collaboration
0.64 institution.mean.norm.impact.factor
0.41 country.germany
−0.67 country.china
−0.61 country.korea
−0.56 last.author.gender.not.found
−0.43 country.japan
NO geo reuse & YES high institution output
0.66 institution.research.output.tr
0.58 institution.harvard
0.46 has.K.funding
0.42 institution.stanford
−0.79 pubmed.is.geo.reuse
−0.62 country.australia
−0.46 institution.rank
NOT animals or mice
0.51 pubmed.is.humans
0.43 pubmed.is.diagnosis
0.40 pubmed.is.effectiveness
−0.93 pubmed.is.animals
−0.86 pubmed.is.mice
Humans & cancer
0.84 pubmed.is.humans
0.75 pubmed.is.cancer
0.67 pubmed.is.cultured.cells
0.52 institution.is.medical
0.47 pubmed.is.core.clinical.journal
−0.68 pubmed.is.plants
−0.49 pubmed.is.fungi
Institution is government & NOT higher ed
0.92 institution.is.govnt
0.70 country.germany
0.65 country.france
0.46 institution.international.collaboration
−0.78 institution.is.higher.ed
−0.56 country.canada
−0.51 institution.stanford
−0.42 institution.is.medical
NO K funding or P funding
0.56 has.R01.funding
0.49 has.R.funding
0.41 num.post2006.morethan500k.tr
0.41 num.post2006.morethan750k.tr
0.40 num.post2006.morethan1000k.tr
−0.65 has.K.funding
−0.63 has.P.funding
Authors prev GEOAE sharing & OA & arry creation
0.83 last.author.num.prev.geoae.sharing.tr
0.74 last.author.num.prev.microarray.creations.tr
0.73 last.author.num.prev.oa.tr
0.60 first.author.num.prev.geoae.sharing.tr
0.47 first.author.num.prev.oa.tr
0.46 first.author.num.prev.microarray.creations.tr
0.40 institution.stanford
−0.44 years.ago.tr
First author num prev pubs & first year pub
0.83 first.author.num.prev.pubs.tr
0.77 first.author.year.first.pub.ago.tr
0.73 first.author.num.prev.pmc.cites.tr
0.52 first.author.num.prev.other.sharing.tr

After imputing missing values, I calculated scores for each of the 15 factors for each of the 11,603 data collection studies.

**Figure 3:**
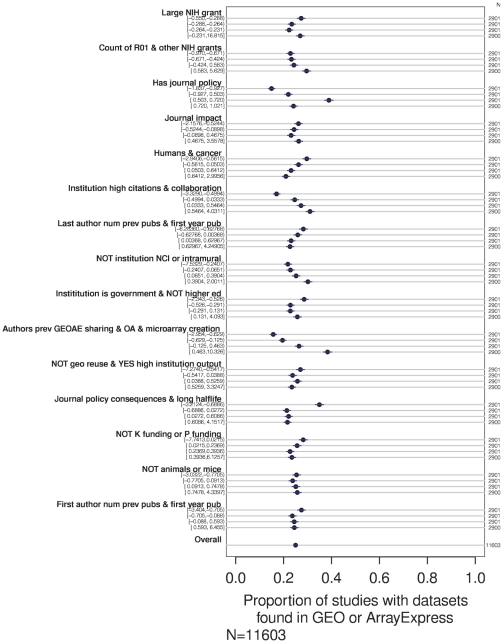
Association between shared data and first-order factors. Percentage of studies with shared data is shown for each quartile for each factor. Univariate analysis.

Many of the factor scores demonstrated a correlation with frequency of data sharing in univariate analysis, as seen in Figure 3. Several factors seemed to have a linear relationship with data sharing across their whole range. For example, whereas the data sharing rate was relatively low for studies with the lowest scores on the factor related to the citation and collaboration rate of the corresponding author's institution (in Figure 3, the first row under the heading “Institution high citation & collaboration”), the data sharing rate was higher for studies that scored within the 25^th^ to 50^th^ percentile on that factor, higher still for studies the third quartile, and studies from highly-cited institutions, above the 75^th^ percentile had a relatively high rate of data sharing. A trend in the opposite direction can be seen for the factor “Humans & cancer”: the higher a study scored on that factor, the less likely it was to have shared its data.

**Figure 4:**
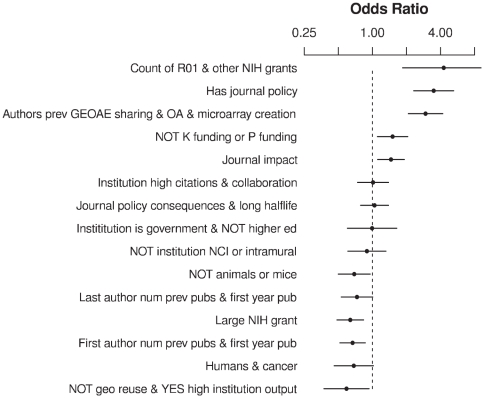
Odds ratios of data sharing for first-order factor, multivariate model. Odd ratios are calculated as factor scores are each varied from their 25^th^ percentile value to their 75^th^ percentile value. Horizontal lines show the 95% confidence intervals of the odds ratios.

Most of these factors were significantly associated with data-sharing behavior in a multivariate logistic regression: p = 0.18 for “Large NIH grant”, p<0.05 for “No GEO reuse & YES high institution output” and “No K funding or P funding”, and p<0.005 for the other first-order factors. The increase in the odds of data sharing is illustrated in Figure 4 as scores on each factor in the model are moved from their 25^th^ percentile value to their 75^th^ percentile value.

### Second-order factors

The heavy correlations between the first-order factors suggested that second-order factors may be illuminating. Scree plot analysis of the correlations between the first-order factors suggested a solution containing five second-order factors. I calculated the factors using a “varimax” rotation to find orthogonal factors. Loadings on the first-order factors are given in Table 2.

**Table 2:** Second-order factor loadings, by first-order factors.

Amount of NIH funding
0.89 Count of R01 & other NIH grants
0.49 Large NIH grant
−0.55 NO K funding or P funding
Cancer & humans
0.83 Humans & cancer
OA journal & previous GEO-AE sharing
0.59 Authors prev GEOAE sharing & OA & microarray creation
0.43 Institution high citations & collaboration
0.31 First author num prev pubs & first year pub
−0.36 Last author num prev pubs & first year pub
Journal impact factor and policy
0.57 Journal impact
0.51 Last author num prev pubs & first year pub
Higher Ed in USA
0.40 NO geo reuse+YES high institution output
−0.44 Institution is government & NOT higher ed

Since interactions make these second-order variables slightly difficult to interpret, I followed the method explained by Gorsuch [51] to calculate the loadings of the second-order variables directly on the original variables. The results are listed in Table 3. I named the second-order factors based on the loadings on the original variables.

**Table 3:** Second-order factor loadings, by original variables.

Amount of NIH funding
0.87 nih.cumulative.years.tr
0.85 num.grants.via.nih.tr
0.84 max.grant.duration.tr
0.82 num.grant.numbers.tr
0.80 pubmed.is.funded.nih
0.79 nih.max.max.dollars.tr
0.70 nih.sum.avg.dollars.tr
0.70 nih.sum.sum.dollars.tr
0.59 has.R.funding
0.59 num.post2003.morethan500k.tr
0.58 country.usa
0.58 has.U.funding
0.57 has.R01.funding
0.55 num.post2003.morethan750k.tr
0.53 has.T.funding
0.53 num.post2003.morethan1000k.tr
0.49 num.post2004.morethan500k.tr
0.45 num.post2004.morethan750k.tr
0.44 has.P.funding
0.43 num.post2004.morethan1000k.tr
0.43 num.nih.is.nci.tr
0.35 num.post2005.morethan500k.tr
0.32 num.nih.is.nigms.tr
0.31 num.post2005.morethan750k.tr
Cancer & humans
0.60 pubmed.is.cancer
0.59 pubmed.is.humans
0.52 pubmed.is.cultured.cells
0.43 pubmed.is.core.clinical.journal
0.39 institution.is.medical
−0.58 pubmed.is.plants
−0.50 pubmed.is.fungi
−0.37 pubmed.is.shared.other
−0.30 pubmed.is.bacteria
OA journal & previous GEO-AE sharing
0.40 first.author.num.prev.geoae.sharing.tr
0.37 pubmed.is.open.access
0.37 first.author.num.prev.oa.tr
0.35 last.author.num.prev.geoae.sharing.tr
0.32 pubmed.is.effectiveness
0.32 last.author.num.prev.oa.tr
0.31 pubmed.is.geo.reuse
−0.38 country.japan
Journal impact factor and policy
0.48 journal.impact.factor.log
0.47 jour.policy.requires.microarray.accession
0.46 jour.policy.mentions.exceptions
0.46 pubmed.num.cites.from.pmc.tr
0.45 journal.5yr.impact.factor.log
0.45 jour.policy.contains.word.miame.mged
0.42 last.author.num.prev.pmc.cites.tr
0.41 jour.policy.requests.accession
0.40 journal.immediacy.index.log
0.40 journal.num.articles.2008.tr
0.39 years.ago.tr
0.36 jour.policy.says.must.deposit
0.35 pubmed.num.cites.from.pmc.per.year
0.33 institution.mean.norm.citation.score
0.32 last.author.year.first.pub.ago.tr
0.31 country.usa
0.31 last.author.num.prev.pubs.tr
0.31 jour.policy.contains.word.microarray
−0.31 pubmed.is.open.access
Higher Ed in USA
0.36 institution.stanford
0.36 institution.is.higher.ed
0.35 country.usa
0.35 has.R.funding
0.33 has.R01.funding
0.30 institution.harvard
−0.37 institution.is.govnt

**Figure 5:**
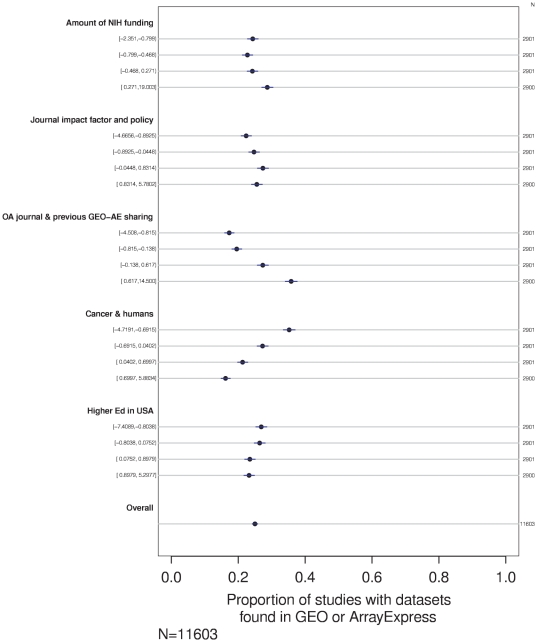
Association between shared data and second-order factors. Percentage of studies with shared data is shown for each quartile for each factor. Univariate analysis.

I then calculated factor scores for each of these second-order factors using the original attributes of the 11,603 datapoints. In univariate analysis, scores on several of the five factors showed a clear linear relationship with data sharing frequency, as illustrated in Figure 5.

**Figure 6:**
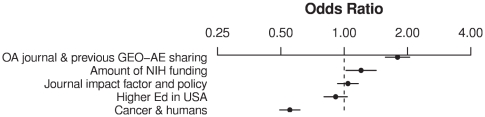
Odds ratios of data sharing for second-order factor, multivariate model. Odd ratios are calculated as factor scores are each varied from their 25^th^ percentile value to their 75^th^ percentile value. Horizontal lines show the 95% confidence intervals of the odds ratios.

All five of the second-order factors were associated with data sharing in multivariate logistic regression, p<0.001.The increase in odds of data sharing is illustrated in Figure 6 as each factor in the model is moved from its 25^th^ percentile value to its 75^th^ percentile value.

Finally, to understand which of these factors was most predictive of data sharing behaviour, I performed supervised hierarchical clustering using the second-order factors. Splits on “OA journal & previous GEO-AE sharing” and “Cancer & Humans” were clearly the most informative, so I simply split these two factors at their medians and looked at the data sharing prevalence. As shown in Table 4, studies that scored high on the “OA journal & previous GEO-AE sharing” factor and low on the “Cancer & Humans” factor were almost three times as likely to share their data as a “Cancer & Humans” study published without a strong “OA journal & previous GEO-AE sharing” environment.

**Table 4:** Data sharing prevalence of subgroups divided at medians of two second-order factors [95% confidence intervals].

number of studies with shared data/ number of studies	Above the median value for the factor “Cancer & Humans”	Below the median value for the factor “Cancer & Humans”	Total
**Above the median value for the factor “OA and previous GEO-AE sharing”**	629/2624 = 24% [22%, 26%]	1184/3178 = **37% [36%, 39%]**	1813/5802 = 31% [30%, 32%]
**Below the median value for the factor “OA and previous GEO-AE sharing”**	440/3178 = **14% [13%, 15%]**	648/2623 = 25% [23%, 26%]	1088/5801 = 19% [18%, 20%]
**Total**	1069/5802 = 18% [17%, 19%]	1832/5801 = 32% [30%, 33%]	**2901/11603 = 25% [24%, 26%]**

## Discussion

This study explored the association between attributes of a published experiment and the probability that its raw dataset was shared in a publicly accessible database. I found that 25% of studies that performed gene expression microarray experiments have deposited their raw research data in a primary public repository. The proportion of studies that shared their gene expression datasets increased over time, from less than 5% in early years, before mature standards and repositories, to 30%–35% in 2007–2009. This suggests that perhaps 45% of recent gene expression studies have made their data available somewhere on the internet, after accounting for datasets overlooked by the automated methods of discovery [44]. This estimate is consistent with a previous manual inventory [15].

Many factors derived from an experiment's topic, impact, funding, publishing, institutional, and authorship environments were associated with the probability of data sharing. In particular, authors publishing in an open access journal, or with a history of sharing and reusing shared gene expression microarray data, were most likely to share their data, and those studying cancer or human subjects were least likely to share.

It is disheartening to discover that human and cancer studies have particularly low rates of data sharing. These data are surely some of the most valuable for reuse, to confirm, refute, inform and advance bench-to-bedside translational research [52] Further studies are required to understand the interplay of an investigator's motivation, opportunity, and ability to share their raw datasets [53], [54]. In the mean time, we can make some guesses: As is appropriate, concerns about privacy of human subjects' data undoubtedly affect a researcher's willingness and ability (perceived or actual) to share raw study data. I do not presume to recommend a proper balance between privacy and the societal benefit of data sharing, but I will emphasize that researchers should assess the degree of re-identification risk on a study-by-study basis [55], evaluate the risks and benefits across the wide range of stakeholder interests [56], and consider an ethical framework to make these difficult decisions [57]. Learning how to make these decisions well is difficult: it is vital that we educate and mentor both new and experienced researchers in best practices. Given the low risk of re-identification through gene expression microarray data (illustrated by its inclusion in the Open-Access Data Tier at http://target.cancer.gov/dataportal/access/policy.asp), data-sharing rates could also be low for reasons other than privacy. Cancer researchers may perceive their field as particularly competitive, or cancer studies may have relatively strong links to industry – two attributes previously associated with data withholding [58], [59].

NIH funding levels were associated with increased prevalence of data sharing, though the overall probability of sharing remains low even in well-funded studies. Data sharing was infrequent even in studies funded by grants clearly covered by the NIH Data Sharing Policy, such as those that received more than one million dollars per year and were awarded or renewed since 2006. This result is consistent with reports that the NIH Data Sharing Policy is often not taken seriously because compliance is not enforced [54]. It is surprising how infrequently the NIH Data Sharing Policy applies to gene expression microarray studies (19% as per a pilot to this study [60]). The NIH may address these issues soon within its renewed commitment to make data more available [61].

I am intrigued that publishing in an open access journal, previously sharing gene expression data, and previously reusing gene expression data were associated with data sharing outcomes. More research is required to understand the drivers behind this association. Does the factor represent an attitude towards “openness” by the decision-making authors? Does the act of sharing data lower the perceived effort of sharing data again? Does it dispel fears induced by possible negative outcomes from sharing data? To what extent does recognizing the value of shared data through data reuse motivate an author to share his or her own datasets?

People often wonder whether the attitude towards data sharing varies with age. Although I was not able to capture author age, I did estimate the number of years since first and last authors had published their first paper. The analysis suggests that first authors with many years in the field are less likely to share data than those with fewer years of experience, but no such association was found for last authors. More work is needed to confirm this finding given the confounding factor of previous data-sharing experience.

Gene expression publications associated with Stanford University have a very high level of data sharing. The true level of open data archiving is actually much higher than that reflected in this study: Stanford University hosts a public microarray repository, and many articles that did not have a dataset link from GEO or ArrayExpress do mention submission to the Stanford Microarray Database. If one were looking for a community on which to model best practices for data sharing adoption, Stanford would be a great place to start.

Similarly, *Physiological Genomics* has very high rates of public archiving relative to other journals. Perhaps not coincidentally, to my knowledge *Physiological Genomics* is the only journal to have published an evaluation of their author's attitudes and experiences following the adoption of new data archiving requirements for gene expression microarray data [21].

Analyzing data sharing through bibliometric and data-mining attributes has several advantages: We can look at a very large set of studies and attributes, our results are not biased by survey response self-selection or reporting bias, and the analysis can be repeated over time with little additional effort.

However, this approach does suffer its own limitations. Filters for identifying microarray creation studies do not have perfect precision, so some non-data-creation studies may be included in the analysis. Because studies that do not create data will not have data deposits, their inclusion alters the composition of what I consider to be studies that create but do not share data. Furthermore, my method for detecting data deposits overlooks data deposits that are missing PubMed identifiers in GEO and ArrayExpress, so the dataset misclassifies some studies that did in fact share their data in these repositories.

I made decisions to facilitate analysis, such as assuming that PubMed identifiers were monotonically increasing with publication date and using the current journal data-sharing policy as a surrogate for the data-sharing policy in place when papers were published. These decisions may have introduced errors.

Missing data may have obscured important information. For example, articles published in journals with policies that I did not examine had a lower rate of data sharing than articles published in journals whose “Instructions to Authors” policies I did quantify. It is likely that a more comprehensive analysis of journal data-sharing policies would provide additional insight. Similarly, the information on funders was limited: I only included funding data on NIH grants. Inclusion of more funders would help us understand the general role of funder policy and funding levels.

Previous work [58] found that investigator gender was correlated with data withholding. It is important to look at gender in multivariate analysis since male scientists are more likely than women to have large NIH grants [62]. Because gender did not contribute heavily to any of the derived factors in this study, additional analysis will be necessary to investigate its association with data sharing behaviour in this dataset. It should be noted that the source of gender data has limitations. The Baby Name Guesser algorithm empirically estimates gender by analyzing popular usage on the internet. Although coverage across names from diverse ethnicities seems quite good, the algorithm is relatively unsuccessful in determining the gender of Asian names. This may have confounded the gender analysis, and the “gender not found” variable might have served as an unexpected proxy for author ethnicity.

The Author-ity system provides accurate author publication histories: A previous evaluation on a different sample found that only 0.5% of publication histories erroneously included more than one author, and about 2% of clusters contained a partial inventory of an author's publication history due to splitting a given author across multiple clusters [46]. However, because the lumping does not occur randomly, my attributes based on author publication histories may have included some bias. For example, the documented tendency of Author-ity to erroneously lump common Japanese names [46] may have confounded the author-history variables with author-ethnicity and thereby influenced the findings on first-author age and experience.

In previous work I used h-index and a-index metrics to measure “author experience” for both the first and last author [60] (in biomedicine, customarily, the first and last authors make the largest contributions to a study and have the most power in publication decisions). A recent paper [63] suggests that a raw count of number of papers and number of citations is functionally equivalent to the h-index and a-index, so I used the raw counts in this study for computational simplicity. Reliance on citations from PubMed Central (to enable scripted data collection) meant that older studies and those published in areas less well represented in PubMed Central were characterized by an artificially low citation count.

The large sample of 11,603 studies captured a fairly diverse and representative subset of gene expression microarray studies, though it is possible that gene expression microarray studies missed by the full-text filter differed in significant ways from those that used mainstream vocabulary to describe their wetlab methods. Selecting a sample based on queries of non-subscription full-text content may have introduced a slight bias towards open access journals. It is worth noting that this study demonstrates the value of open access and open full-text resources for research evaluation.

In regression studies it is important to remember that associations do not imply causation. It is possible, for example, that receiving a high level of NIH funding and deciding to share data are not causally related, but rather result from the exposure and excitement inherent in a “hot” subfield of study.

Importantly, this study did not consider directed sharing, such as peer-to-peer data exchange or sharing within a defined collaboration network, and thus underestimates the amount of data sharing in all its forms. Furthermore, this study underestimated public sharing of gene expression data on the Internet. It did not recognize data listed in journal supplementary information, on lab or personal web sites, or in institutional or specialized repositories (including the well-regarded and well-populated Stanford Microarray Database). Finally, the study methods did not recognize deposits into the Gene Expression Omnibus or ArrayExpress unless the database entry was accompanied by a citation to the research paper, complete with PubMed identifier.

Due to these limitations, care should be taken in interpreting the estimated levels of absolute data sharing and the data-sharing status of any particular study listed in the raw data. More research is needed to attain a deep understanding of information behaviour around research data sharing, its costs and benefits to science, society and individual investigators, and what makes for effective policy.

That said, the results presented here argue for action. Even in a field with mature policies, repositories and standards, research data sharing levels are low and increasing only slowly, and data is least available in areas where it could make the biggest impact. Let's learn from those with high rates of sharing and work to embrace the full potential of our research output.

### Availability of dataset, statistical scripts, and data collection source code

Raw data and statistical scripts are available in the Dryad data repository at doi:10.5061/dryad.mf1sd [64]. Data collection source code is available at http://github.com/hpiwowar/pypub.

## Supporting Information

Figure S1 **Associations between shared data and author attribute variables.** Percentage of studies with shared data is shown for each quartile for continuous variables.
(EPS)

Figure S2 **Associations between shared data and journal attribute variables.** Percentage of studies with shared data is shown for each quartile for continuous variables.
(EPS)

Figure S3 **Associations between shared data and study attribute variables.** Percentage of studies with shared data is shown for each quartile for continuous variables.
(EPS)

Figure S4 **Associations between shared data and funding attribute variables.** Percentage of studies with shared data is shown for each quartile for continuous variables.
(EPS)

Figure S5 **Associations between shared data and country and institution attribute variables.** Percentage of studies with shared data is shown for each quartile for continuous variables.
(EPS)

Table S1 **Structure matrix with correlations between all attributes and first-order factors.**
(TXT)
